# Hypoxia and oxidative stress markers in pediatric patients undergoing hemodialysis: cross section study

**DOI:** 10.1186/1471-2369-13-136

**Published:** 2012-10-13

**Authors:** Enas A Hamed, Taghrid B El-Abaseri, Amany O Mohamed, Ahmed R Ahmed, Tarek H El-Metwally

**Affiliations:** 1 Departments of Medical Physiology, Assiut University, Assiut, Egypt; 2 School of Medicine, Medical Biochemistry Department, Suez Canal University, Ismailia, Egypt; 3 Departments of Medical Biochemistry, Assiut University, Assiut, Egypt; 4 Departments of Pediatrics, Faculty of Medicine, Assiut University, Assiut, Egypt

## Abstract

**Background:**

Tissue injury due to hypoxia and/or free radicals is common in a variety of disease processes. This cross-sectional study aimed to investigate effect of chronic kidney diseases (CKD) and hemodialysis (HD) on hypoxia and oxidative stress biomarkers.

**Methods:**

Forty pediatric patients with CKD on HD and 20 healthy children were recruited. Plasma hypoxia induced factor-1α (HIF-1α), vascular endothelial growth factor (VEGF) were measured by specific ELISA kits while, total antioxidant capacity (TAC), total peroxide (TPX), pyruvate and lactate by enzymatic/chemical colorimetric methods. Oxidative stress index (OSI) and lactate/pyruvate (L/P) ratio were calculated.

**Results:**

TAC was significantly lower while TPX, OSI and VEGF were higher in patients at before- and after-dialysis session than controls. Lactate and HIF-1α levels were significantly higher at before-dialysis session than controls. Before dialysis, TAC and L/P ratio were lower than after-dialysis. In before-dialysis session, VEGF correlated positively with pyruvate, HIF-1α and OSI correlated positively with TPX, but, negatively with TAC. In after-dialysis session, HIF-1α correlated negatively with TPX and OSI; while, OSI correlated positively with TPX.

**Conclusions:**

CKD patients succumb considerable tissue hypoxia with oxidative stress. Hemodialysis ameliorated hypoxia but lowered antioxidants as evidenced by decreased levels of HIF-1α and TAC at before- compared to after-dialysis levels.

## Background

In chronic kidney diseases (CKD), as a worldwide public health problem, there is a functional loss of renal glomeruli caused by glomerular or interstitial renal diseases [1]. Endothelial dysfunction and cardiovascular disease are the major cause of mortality in patients on maintenance hemodialysis (HD) and peritoneal dialysis (PD) [2]. Endothelial dysfunction could be a consequence of accumulation of uremic toxins functioning as inhibitors of endothelial function (Cross et al., 2001). Pathogenetic role for impaired vasculogenesis and reduction in peritubular capillary density in the progression of CKD was demonstrated in animal models and human studies [3].

Hypoxia is a state of reduced oxygen pressure below a critical threshold, which restricts the function of organs, tissues and cells. A decrease in oxygen tensions of the kidney was demonstrated in a number of experimental models of CKD. This led to the broad recognition that chronic cellular hypoxia of the kidney is the final common pathway in the progression of CKD leading to eventual kidney failure [4]. A group of transcription factors, designated hypoxia inducible transcription factors, are specifically induced by low tissue oxygen tension and are likely to have a role in the oxygen-sensing mechanism and reparative reaction. Hypoxia induced factor-1 (HIF-1) is a heterodimeric protein consisting of an alpha subunit (with 3 isoforms -1α, -2α and -3α), and a beta subunit (also known as the aryl hydrocarbon receptor nuclear translocator, ARNT), which is constitutively expressed [5]. In normoxia, the regulatory HIF-1α subunit is hydroxylated on two prolines by iron dependent prolyl-hydroxylases to be degraded through the ubiquitin-proteasome pathway - via its interaction with the von Hippel-Lindau tumor suppressor protein. Under cellular hypoxia, inhibition of such hydroxylases spares HIF-1α that translocates into nucleus. There, it heterodimerizes with HIF-1β and binds to the hypoxic response elements of target gene regulatory sequences. This induces the transcription of genes implicated in the control of metabolism and angiogenesis, as well as apoptosis and cellular stress response [6].

Genes activated by HIF can be schematically classified into three functional groups that could halt CKD progression. (1) Proteins participating in erythropoiesis, thereby increasing tissue oxygen delivery, e.g., erythropoietin, transferrin, transferrin receptor, heme oxygenase-1. (2) Proteins that increase local oxygen delivery to tissues, e.g., inducible nitric oxide synthesis and vascular endothelial growth factor (VEGF). (3) Proteins required for adaptation to anaerobic cellular metabolism: glucose transporter-1 and most glycolytic enzymes [7].

VEGF, a 35kDa molecule, is a chemoattractant that enables the homing of regenerative endothelial cells into local vascular lesions [8]. In the aging kidneys, loss of VEGF expression in podocytes was associated with the reduced number of endothelial progenitor cells [9]. However, there is still a considerable debate over the vasculoprotective vs. pro-inflammatory effects of VEGF [10]. VEGF is known to play multiple roles in normal renal physiology and renal disorders. In the kidney, VEGF is expressed in the visceral epithelial cells of glomeruli, proximal and distal convoluted tubules and can induce nephrogenesis and vasculogenesis. It promotes cellular proliferation, differentiation, and survival and contributes to interstitial matrix remodeling [11].

Oxidative stress is an imbalanced generation of reactive oxygen species (ROS) vs. the counteracting antioxidants. In CKD, such stress is multifactorial. Uremic toxins, interactions between circulating mononuclear cells and bioincompatible dialyzers or contaminated dialysate, and infections, can act as triggers [12]. In addition, dialysis patients have reduced levels of vitamins C and E, selenium, and β-carotene, and they show lowered antioxidant enzyme activities; paraoxonase, catalase, superoxide dismutase, heme oxygenase and glutathione peroxidase [13]. Although increased oxidative stress is increasingly recognized as an important metabolic event in adults with CKD [14], there are few studies utilizing global biomarkers of oxidative stress in children with CKD [15,16].

To our knowledge, there are a few studies addressing systemic oxidative stress and cellular hypoxia biomarkers together in children with CKD on regular hemodialysis. We suggested that impaired response to tissue hypoxia and oxidative stress would be major determinants to clinical outcomes in such patients. This cross-sectional study aimed at assessing the effect of CKD as well as maintenance HD on plasma oxidative stress markers [total anti-oxidant capacity (TAC), total peroxide (TPX) and oxidative stress index (OSI)] and standard hypoxia biomarkers [lactate, pyruvate and lactate/pyruvate (L/P) ratio, and VEGF] as well as HIF-1α in CKD pediatric patients before and after HD session. HIF-1α, the key regulatory cellular hypoxia effector is investigated for the first-time to our knowledge as a plasma biomarker. The relationship among these markers will be statistically analyzed.

## Methods

### Study groups

Forty children and adolescents with CKD (25 male and 15 female) on maintenance HD with an age range of 6–15 years [12.83±1.89 years, mean+/− standard deviation (SD)] were voluntarily recruited to this cross section study at the Pediatric Nephrology and Dialysis Unit, Assiut Children University Hospital, Assiut, Egypt, during the period from January 2010 to September 2010. At the time of the study, all the patients were on regular three HD sessions per week; each time for 3–4 h (total 12 h weekly) for more than 3 months with polysulfone dialyzing membranes, after creatinine clearance had fallen below 8–12 mL/min and/or pharmacological treatment and diet had proved inadequate to control clinical symptoms. The mean dialysis duration was 2.18±1.36 years (range: 1–7 years). All patients were on dietary protein restriction, 1 g/kg body weight per day. The dialysate used was a standard ionic composition and bicarbonate-based buffer (Na^+^ 138 mmol/L, HCO_3_^-^ 36.6 mmol/L, K^+^ 1.5 mmol/L, Ca^2+^ 1.25 to 1.75 mmol/L, Mg^2+^ 0.75 mmol/L) for all cases. The blood flow was 120–150 mL/min and dialysis fluid was used at a flow rate of 240–300 mL/min. Vascular access was arteriovenous fistula in the upper limbs. The causes of CKD were: urolithiasis (*n*= 11), recurrent pyelonephritis with vesico-ureteral reflux (*n*= 8), chronic glomerulopathies (*n* = 16), hypoplastic-dysplastic kidney (*n* = 2), posterior urethral valve (*n*= 2) and cystinosis (*n*= 1). The healthy control group consisted of 20 age- and sex-matching volunteers aging 6–15 years (12.10±2.59 years) from those admitted for routine check-up.

At the time of entry, none of the participants was receiving non-steroidal anti-inflammatory drugs, immunosuppressive therapy or any other drugs that might have interfered with the oxidative stress, and none had uncontrolled hypertension or a history of seizure. Patients with infections, vasculitides, or respiratory, metabolic and hepatic diseases were excluded. Control subjects were on a normal diet without vitamin supplementation and were free from any infection or medication. Routine analyses showed that controls had normal renal function tests and no disorders of lipid metabolism. Both patients and controls were nonsmokers. The study was approved by the local ethics committee and was conducted in accordance with the Declaration of Helsinki and informed consent was obtained in every case from their legal guardians. Demographic, clinical and biochemical characteristics of all participants were reported.

### Laboratory investigations

Blood samples (5 mL) with and without EDTA/sodium fluoride as anticoagulant were obtained via venipuncture after the participants had fasted overnight (between 8.30 P.M. and 9.30 A.M.), and serum and plasma were separated and aliquot frozen at −80°C till used. In HD patients, venous blood samples were drawn immediately before and after hemodialysis session. Baseline laboratory investigations were carried out for all patients and controls including complete blood count, serum urea and creatinine, arterial pH, arterial blood gases and infection screening, which included blood and urinary cultures by standard methods. Arterial pH, PaO_2_, PaCO_2_, HCO_3_^-^ were determined using a pH/blood gas analyzer on anaerobically collected blood drawn in ice-cold, heparinized glass syringes.

ELISA assays were utilized to measure each of VEGF (Cat# ELH-VEGF-001, RayBiotech, Inc, Norcross GA 30092 - lower detection limit is 10 pg/mL) and HIF-1α (Cat# DYC1935-5, R&D Systems, Minneapolis, MN 55413, USA - lower detection limit is 3 pg/mL) with specific capture and biotinylated detection antibodies and Streptavidin-Horse Radish Peroxidase/tetramethylbenzidine as the detection system. Total antioxidant capacity was estimated according to the colorimetric method of Koracevic et al. [17] with uric acid as standard. Total plasma peroxide concentrations were determined using the colorimetric FOX2 method with H_2_O_2_ standard as modified by Harma et al. [18]. Briefly, 250 μM FOX2 reagent (9.8 mg ammonium ferrous sulfate in 10 mL of 250 mmol H_2_SO_4_ was added to 90 mL HPLC-grade methanol containing 79.2 mg butylated hydroxytoluene, finally, 7.6 mg xylenol orange was added. The blank reagent is devoted of ferrous sulfate. 200 μL of plasma was incubated with 1.8 mL FOX2 or blank reagent at room temperature for 30 min. Tubes were centrifuged at 12,000g for 10 min and absorbance of clear supernatant was determined at 560 nm vs. blank. The coefficient of variation for individual plasma samples was less than 5%. Percent ratio of TPX to TAC level is the oxidative stress index (OSI; = TPX, μmol/L/TAC, μmol/L X 100) [18,19]. Pyruvate was measured following the decrease in UV-absorbance of NADH.H^+^ according to instructions of the manufacturer (Cat# 180 000, Greiner Diagnostics GmbH, D-79353 Bahlingen, Germany). Lactate was colorimetrically measured using lactate oxidase/peroxidase/tribromo-hydroxybenzoic acid-4-aminoantipyrine according to instructions of the manufacturer (Cat# 274 001, Spectrum Biodiagnostics, Cairo, Egypt). Lactate/pyruvate ratio was calculated.

### Data analysis

Statistical Science for Social Package (SPSS Inc, USA) software computer program version 12 was used for data analysis. Data were presented as mean -/+ SD and minimum - maximum or number and percentage (n, %) as appropriate. For comparison of two groups the nonparametric test for independent variables and paired and unpaired student “t” test for parametric variables were used while, comparisons of multiple groups were done using one-way analysis of variation for parametric variables. Spearman’s correlation test was used for parametric variables within each group. For all tests, a probability (*P)* <0.05 was considered significant.

## Results

Table 1 showed relevant participants’ characteristics. Compare to healthy controls, hemoglobin, pH, PCO_2_ and HCO_3_^-^ were significantly higher (*P* <0.001) while, creatinine, urea and PCO_2_ were significantly lower (*P* <0.001) than patients.

**Table 1:** Demographic and clinical characteristics of studied groups

**Parameters**	**Controls(*n *= 20)**	**Patients(*n *= 40)**	**Significance *P *<**
**Age** (years)	12.10 ± 2.59	12.83 ± 1.89	0.126
	6.00 - 15.00	6.00 - 15.00	
**Gender**			
Male	10 (50.00%)	25 (62.50%)	
Female	10 (50.00%)	15 (37.50%)	
**Time on hemodialysis** (years)	-	2.18 ± 1.36	-
		1.00 - 7.00	
**Hemoglobin** (gram/dL)	11.50 ± 0.94	9.35 ± 0.85	**0.001**
	10.00 - 12.90	8.70 - 11.60	
**Creatinine** (μmol/L)	93.6 ± 22.41	468.00 ± 177.55	**0.001**
	58.00 - 127.00	157.00 - 750.00	
**Urea** (mg/dL)	22.90 ± 5.64	196.10 ± 23.50	**0.001**
	15.00 - 32.00	150.00 - 229.00	
**pH**	7.39 ± 0.02	7.32 ± 0.02	**0.001**
	7.35 - 7.40	7.30 - 7.35	
**PCO**_**2**_ (mmHg)	38.95 ± 1.10	31.74 ± 6.92	**0.001**
	37.00 - 40.00	18.20 - 43.10	
**PO**_**2**_ (mmHg)	87.59 ± 11.5	112.56 ± 24.35	**0.001**
	88.00 - 96.00	87.00 - 169.00	
**HCO**_**3**_^**-**^ (μmol/L)	22.90 ± 0.64	15.66 ± 4.13	**0.001**
	22.00 - 24.00	8.10 - 21.20	

Data are express as mean ± SD minimum - maximum or number (%); significance versus controls.

Total antioxidant capacity was significantly lower while TPX, OSI and VEGF were significantly higher in patients at before- and after-dialysis session than healthy controls. Lactate and HIF-1α levels were significantly higher (*P* <0.019, *P* <0.002) at before-dialysis session compared to healthy controls. Before dialysis, TAC and L/P ratio were higher (*P* <0.036, *P* <0.001) compared to after-dialysis level (Table 2).

**Table 2:** Changes in the plasma levels of the investigated cellular hypoxia and oxidative stress biomarkers in studied groups

**Parameters**	**Controls(n = 20)**	**Before dialysis(n = 40)**	**After dialysis(n = 40)**
**Total antioxidant capacity** (mmol/L)	0.66 ± 0.05	0.60 ± 0.05	0.57 ± 0.06
		*P <0.001	*P <0.001
			**P <0.036
**Total peroxides** (μmol/L)	30.50 ± 12.76	38.50 ± 12.52	37.25 ± 12.92
		*P <0.021	*P <0.050
			**P = 0.673
**Oxidative stress index**	4.63 ± 1.89	6.52 ± 2.30	6.53 ± 2.23
		*P <0.002	*P <0.002
			**P = 0.983
**Lactate** (mg/dL)	12.06 ± 3.66	15.90 ± 6.56	13.34 ± 5.99
		*P <0.019	*P = 0.425
			**P = 0.061
**Pyruvate** (mg/dL)	5.02 ± 3.67	6.47 ± 8.69	6.84 ± 4.02
		*P = 0.402	*P = 0.280
			**P = 0.777
**Lactate/pyruvate ratio**	3.36 ± 1.69	4.07 ± 2.70	2.57 ± 1.76
		*P = 0.234	*P = 0.187
			**P <0.007
**Vascular endothelial growth factor** (pg/mL)	4.00 ± 1.26	32.91 ± 24.40	33.15±24.58
		*P <0.001	*P <0.001
			**P =0.964
**Hypoxia-induced factor- 1α** (pg/mL)	29.45 ± 10.07	57.02 ± 41.86	43.29 ± 26.67
		*P <0.002	*P = 0.115
			**P = 0.120

Data shown are mean ± SD mean -/+ SD (minimum - maximum); *P: significance vs. healthy controls; **P: significance vs. before-dialysis session.

At before-dialysis session, duration of disease positively correlated with HIP-1α (r = 0.677, *P* <0.001) but negatively correlated with VEGF (r = −0.486, *P* < 0.001); VEGF positively correlated with each of pyruvate (r = 0.316, *P* <0.047) and HIF-1α (r = 0.374, *P* <0.018), and, OSI positively correlated with TPX (r = 0.969, *P* <0.001), but, negatively correlated with TAC (r = −0.469, *P* <0.002). At after-dialysis session, HIF-1α negatively correlated with each of TPX (r = −0.529, *P* <0.001) and OSI (r = −0.459, *P* <0.003); while, OSI positively correlated with TPX (r = 0.944, *P* <0.001) (Table 3).

**Table 3:** Correlations among the measured parameters in CKD patients at before- and after-dialysis settings

**Parameters**	**Duration**	**Lactate**	**Pyruvate**	**Lactate/Pyruvate ratio**	**HIF-1α**	**VEGF**	**TAC**	**Total peroxides**
**Lactate**								
Before dialysis	−0.208(0.197)							
After dialysis	−0.184(0.256)							
**Pyruvate**								
Before dialysis	−0.193(0.234)	0.282(0.078)						
After dialysis	−0.095(0.580)	**0.533(0.001)**						
**Lactate/pyruvate ratio**								
Before dialysis	−0.067(0.640)	**0.513(0.001)**	**−0.457(0.003)**					
After dialysis	−0.037(0.824)	0.212(0.188)	**−0.630(0.001)**					
**HIF-1α**								
Before dialysis	**0.677 (0.001)**	0.125(0.442)	−0.149(0.359)	0.220(0.173)				
After dialysis	−0.186(0.249)	0.048(0.770)	0.015(0.929)	0.229(0.156)				
**VEGF**								
Before dialysis	**−0.486(0.001)**	0.291(0.069)	**0.316(0.047)**	0.027(0.866)	**0.374(0.018)**			
After dialysis	0.189 (0.243)	0.125 (0.441)	0.059(0718)	0.098(0.545)	−0.163(0.314)			
**TAC**								
Before dialysis	0.265 (0.098)	−0.174(0.283)	−0.147(0.366)	−0.142(0.384)	0.068(0.676)	−0.206(0.202)		
After dialysis	0.022 (0.894)	0.048(0.771)	−0.148(0.361)	0.105(0.517)	−0.220(0.173)	−0147(0.366)		
**Total peroxides**								
Before dialysis	−0.017(0.918)	−0.032(0.843)	−0.194(0.230)	0.128(0.430)	−0.138(0.394)	0.229(0.156)	−0.247(0.124)	
After dialysis	0.283 (0.077)	0.036(0.825)	0.070(0.668)	−0.204(0.207)	**−0.529(0.001)**	−0.006(0.972)	0.168(0.301)	
**Oxidative stress index**								
Before dialysis	−0.093(0.566)	−0.002(0.989)	0.161(0.322)	0.156(0.337)	−0.172(0.287)	0.245(0.128)	**−0.469(0.002)**	**0.969(0.001)**
After dialysis	0.285 (0.075)	0.015(0.929)	0.113(0.489)	−0.240(0.136)	**−0.459(0.003)**	0.062(0.705)	−0.155(0.340)	**0.944(0.001)**

Data shown are r value (*P*< value). HIF-1α= hypoxia induced factor-1α, VEGF= vascular endothelial growth factor. TAC = total antioxidant capacity.

## Discussion

Relative hypoxia, as the major activator of hypoxia-inducible factor, is detectable in chronic kidney disease tissues irrespective of etiology and is thought to result from a combination of structural and functional changes that include; decreased peritubular blood flow associated with glomerular injury, capillary rarefaction, vasoconstriction, luminal narrowing of atherosclerotic vessels, increased oxygen demand from hyperfiltration and tubular hypertrophy, limited oxygen diffusion as a consequence of extracellular matrix expansion, and renal anemia [20]. Chronically ill patients on HD exist in unique situation because their survival is dependent upon treatments which are operational only 12–18 h per week in six hour sessions. This procedure subjects these patients to innumerable abrupt alterations in the internal environment, especially rapid shifts in pH [21]. In this study, the before-dialysis levels of plasma lactate, HIF-1α and VEGF were significantly higher compared to healthy controls while, lactate/pyruvate ratio was significantly higher compared to after-dialysis level. The lactate level tended to fall with dialysis, at least in part attributable to this procedure. Also, VEGF plasma level was significantly elevated in after-dialysis session compared to healthy controls. Hypoxia is accompanied by a significant increase in blood lactate and severe systemic acidosis as a direct effect of anaerobic metabolism. Beside the direct effects of anaerobic metabolism, catecholamine-induced stimulation of cellular glycolysis and subsequent synthesis of lactate worsens the increased systemic lactate. In such conditions, accumulated pyruvate is metabolized into lactate. Determination of the lactate/pyruvate ratio thus provides a more accurate statement of tissue metabolism and the cytosolic redox condition [22]. As a correction measure, hypoxia-induced HIF-1 upregulates the expression of monocarboxylate transporter 4, which mediates lactic acid efflux, and of membrane-bound carbonic anhydrase IX, which catalyzes the conversion of extracellular CO_2_ to carbonic acid. The latter contributes to the acidification of the extracellular space and enables an increase in intracellular pH through the subsequent uptake of HCO_3_^-^ (a weak base). HIF-1 directly activates the expression of several pro-angiogenic factors, the best characterized of which is VEGF. This event promotes the formation of new blood vessels, thus restoring the supply of oxygen and nutrients [23]. Increased HIF expression has been found in animal models of CKD and in renal biopsy material from patients with diabetic nephropathy and other forms of renal disease [20,24,25].

In this study, HIF-1α was investigated as a plasma biomarker for the first-time world-wide particularly in CKD. At before-dialysis session, plasma VEGF positively correlated with each of pyruvate, and HIF-1α. The positive correlation between HIF-1α and VEGF is expected as VEGF gene is HIF-1α-inducable to increase local oxygen delivery to tissues. This indicates the utility of HIF-1α as plasma biomarker of cellular hypoxia in CKD that reflects the disease outcomes. However, a reparative role for the induced HIF-1 in CKD is questionable in light of blockade of the induction of erythropoietin by the accumulating uremic toxins [11,26-28].

The role of ROS and/or decreased antioxidant activity in the development of CKD complications, such as atherosclerosis and related cardiovascular disturbances is well-established [14]. In this study children with CKD showed increased plasma TPX and OSI and decreased TAC levels at both of before- and after-dialysis compared to healthy controls. Increased oxidative stress has been reported in numerous adult studies in patients with CKD [29]. However, there are few reports on the anti-oxidant system in children with CKD [15,30,31]. Zwolińska et al. [15] reported increased lipid peroxidation, monitored by plasma and erythrocyte malondialdehyde (MDA), together with decreased superoxide dismutase, catalase and glutathione peroxidase activities in children with chronic renal failure before-dialysis as compared to healthy age-matched subjects. The results of our study show significant decreased in TAC levels in after-dialysis compared to before-dialysis which may be related to the loss of antioxidants during dialysis. Turi et al. [32] observed an increase of erythrocytic-MDA in HD children. Daschner et al. [33] showed high plasma MDA levels in a group of ten pediatric patients on HD and in eleven children on peritoneal dialysis. Other studies indicate an impairment of antioxidant systems and augmentation of oxidants during HD sessions in adult patients with end stage renal diseases [34-36].

One reason for oxidative stress in patients with renal failure is the underlying disease itself. Renal toxicity and immunological disorders of the kidney result in an elevated formation of ROS which is active in the pathogenesis of kidney disease. However, treatment procedures were also shown to induce oxidative stress. During HD, incomplete correction of the uremic toxicity together with the untoward effects of dialysis, malnutrition and the progressive worsening of clinical condition, can lead to oxidative stress. The later is caused by an abnormal production of oxidants including ROS and uremic toxins with pro-oxidant function correlating defective antioxidant protection. Bioincompatibility of dialysis membranes represents an important source of ROS. Losses of antioxidants via dialysis are the factors that may be responsible for the imbalance between pro-oxidative and antioxidative mechanisms in HD patients [37]. Total antioxidant capacity in renal failure group is diminished to a great extent due to antioxidant exhaustion and inhibition [38]. Oxidative stress can also accelerate the apoptosis of leukocytes in HD patients [39]. The activation of neutrophils and the complement pathway during HD session as the result of interactions of the blood with the dialysis membrane and endotoxin contaminated dialysate, iron overload, the presence of advanced glycation end products, high homocysteine levels, intradialytic cytokine activation, among others, could play a role [40].

However, Drai et al. [41] reported that chronic renal disease patients admitted to HD, presented enhanced levels of MDA and oxidized glutathione, and decreased concentrations of glutathione and glutathione peroxidase, without any changes in plasma levels of TAC, vitamins A and E. Another clinical study reported that TAC measured as Trolox equivalents was higher in HD patients compared with control group. Although MDA and 4-hydroxynonenal levels were also increased, plasma thiols were lower and α-tocopherol was not altered [42]. After HD, plasma levels of thiols, MDA, 4-hydroxynonenal and TAC were normalized. Similar results were found by Samouilidou and Grapsa [43]. Difference in the biomarker used and dietary antioxidant supplementations could be a major cause of such discrepancy.

In our healthy controls, L/P ratio positively correlated TAC; HIF-1α positively correlated with each of L/P ratio and TAC; VEGF positively correlated with each of TPX and OSI. At after-dialysis, HIF-1α negatively correlated with each of TPX and OSI. In this respect, several reports showed an inverse correlation between MDA serum concentration and hemoglobin in the blood of HD patients [29,31]. A recent report connected induced HIF-1α and increases in reactive O_2_ species [44]. The accelerated LPO at the low Hb level might be explained by oxidative stress due to the anemic condition itself. Anemic patients showed an increased frequency of ventilation at peak exercise because of the limited oxygen transport capacity, implying anaerobic metabolism due to hypoxemia and ischemia. It is possible to suggest that hypoxic patients showing high HIF-1α would have good prognosis since their cellular response to hypoxia is functional - providing that downstream effectors such as VEGF and erythropoietin are activated.

## Conclusion

There is considerable cellular hypoxia in pediatric patients with CKD that leads to oxidative stress. HD leads to decrease hypoxia and increase loss of antioxidants as evidenced by decreased levels of HIF-1α and TAC at before- compared to after-dialysis settings. The resulting oxidative stress could contribute to the long-term complications in uremic patients. In compensation, VEGF levels increases to improve oxygen delivery to hypoxic tissue. Further longitudinal studies are necessary to establish the relationship between plasma markers of hypoxia and oxidative stress and clinical outcomes of CKD on maintained HD - particularly utilizing interventions such as antioxidant supplements and improved aeriation. Longitudinal studies are mandatory since preliminary data showed that low blood gases and hemoglobin correlating with high hypoxia biomarkers are fatal.

### Limitation of the study

Our study has several limitations that necessitate further investigations. By nature, a cross-sectional study would not derive a cause/effect conclusion among the investigated parameters and CKD and/or HD. Second, blood gases, pH and bicarbonate were analyzed before dialysis only because of local limitations. Third, the limited number of participants did not allow statistically meaningful subgrouping according to underlying cause of CDK.

## Competing interests

The authors declare that they have no competing interests.

## Authors’ contributions

The authors EAH, TBA, AOM, ARA and THM have made substantial contributions to the concept and design of the study, the acquisition, analysis and interpretation of data, and in drafting the manuscript or critically revising it for important intellectual content. The authors TBA, AOM and THM measured the estimated biochemical parameters and ARA collected the data sheets and blood samples from patients. The authors have also given final approval of the version to be published. Each author has participated sufficiently in the work to take public responsibility for appropriate sections of the content. All authors read and approved the final manuscript.

## Pre-publication history

The pre-publication history for this paper can be accessed here:

http://www.biomedcentral.com/1471-2369/13/136/prepub

## References

[B1] Levey AS Coresh J Balk E Kausz AT Levin A Steffes MW Hogg RJ Perrone RD Lau J Eknoyan G National Kidney Foundation National Kidney Foundation clinical practice guidelines for chronic kidney disease: evaluation, classification, and stratification J Ann Intern Med 2003 139 2 137 147 10.7326/0003-4819-139-2-200307150-00013 12859163

[B2] van Guldener C Lambert J Janssen MJ Donker AJ Stehouwer CD Endothelium-dependent vasodilatation and distensibility of large arteries in chronic haemodialysis patients Nephrol Dial Transplant 1997 12 2 14 18 9269693

[B3] Ohashi R Shimizu A Masuda Y Kitamura H Ishizaki M Sugisaki Y Yamanaka N Peritubular capillary regression during the progression of experimental obstructive nephropathy J Am Soc Nephrol 2002 13 7 1795 1805 10.1097/01.ASN.0000018408.51388.57 12089375

[B4] Yu X Fang Y Liu H Zhu J Zou J Xu X Jiang S Ding X The balance of beneficial and deleterious effects of hypoxia-inducible factor activation by prolyl hydroxylase inhibitor in rat remnant kidney depends on the timing of administration Nephrol Dial Transplant 2012 27 8 3110 3119 10.1093/ndt/gfr754 22399494

[B5] Wenger RH Mammalian oxygen sensing, signaling and gene regulation J Exp Biol 2000 203 Pt 8 1253 1263 10729275 10.1242/jeb.203.8.1253

[B6] Poellinger L Johnson RS HIF-1 and hypoxic response: the plot thickens Curr Opin Genet Dev 2004 14 1 81 85 10.1016/j.gde.2003.12.006 15108809

[B7] Peyssonnaux C Nizet V Johnson RS Role of the hypoxia inducible factors HIF in iron metabolism Cell Cycle 2008 7 1 28 32 10.4161/cc.7.1.5145 18212530

[B8] Young PP Hofling AA Sands MS VEGF increases engraftment of bone marrow‑derived endothelial progenitor cells (EPCs) into vasculature of newborn murine recipients Proc Natl Acad Sci USA 2002 99 18 11951 11956 10.1073/pnas.182215799 12195016 PMC129375

[B9] Kang DH Anderson S Kim YG Mazzalli M Suga S Jefferson JA Gordon KL Oyama TT Hughes J Hugo C Kerjaschki D Schreiner GF Johnson RJ Impaired angiogenesis in the aging kidney: vascular endothelial growth factor and thrombospondin‑1 in renal disease Am J Kidney Dis 2001 37 3 601 611 10.1053/ajkd.2001.22087 11228186

[B10] Zhao Q Ishibashi M Hiasa K Tan C Takeshita A Egashira K Essential role of vascular endothelial growth factor in angiotensin II-induced vascular inflammation and remodeling Hypertension 2004 44 3 264 270 10.1161/01.HYP.0000138688.78906.6b 15262905

[B11] Gunaratnam L Bonventre JV HIF in kidney disease and development J Am Soc Nephrol 2009 20 9 1877 1887 10.1681/ASN.2008070804 19118148

[B12] Kaysen GA Eiserich JP Characteristics and effects of inflammation in end-stage renal disease Semin Dial 2003 16 6 438 446 10.1046/j.1525-139X.2003.16096.x 14629602

[B13] Ece A Gurkan F Kervancioglu M Kocamaz H Gunes A Atamer Y Selek S Oxidative stress, inflammation and early cardiovascular damage in children with chronic renal failure Pediatr Nephrol 2006 21 545 552 10.1007/s00467-006-0039-0 16520949

[B14] Vaziri ND Dicus M Ho ND Boroujerdi-Rad L Sindhu RK Oxidative stress and dysregulation of superoxide dismutase and NADPH oxidase in renal insufficiency Kidney Int 2003 63 1 179 185 10.1046/j.1523-1755.2003.00702.x 12472781

[B15] Zwolińska D Grzeszczak W Kiliś-Pstrusińska K Szprynger K Szczepańska M Lipid peroxidation and antioxidant enzymes in children with chronic renal failure Pediatr Nephrol 2004 19 8 888 892 10.1007/s00467-004-1512-2 15179570

[B16] Zachwieja J Zaniew M Bobkowski W Stefaniak E Warzywoda A Ostalska-Nowicka D Dobrowolska-Zachwieja A Lewandowska-Stachowiak M Siwinska A Beneficial in vitro effect of N-acetyl-cysteine on oxidative stress and apoptosis Pediatr Nephrol 2005 20 725 731 10.1007/s00467-004-1806-4 15809833

[B17] Koracevic D Koracevic G Djordjevic V Andrejevic S Cosic V Method for the measurement of antioxidant activity in human fluids J Clin Pathol 2001 54 5 356 361 10.1136/jcp.54.5.356 11328833 PMC1731414

[B18] Harma M Harma M Erel O Increased oxidative stress in patients with hydatidiform mole Swiss Med Wkly 2003 133 41–42 563 566 14691728 10.4414/smw.2003.10397

[B19] Harma M Harma M Erel O Measurement of the total antioxidant response in preeclampsia with a novel automated method Euro J Obst Gyn Reprod Biol 2005 118 1 47 51 10.1016/j.ejogrb.2004.04.012 15596272

[B20] Fine LG Norman JT Chronic hypoxia as a mechanism of progression of chronic kidney diseases: from hypothesis to novel therapeutics Kidney Int 2008 74 7 867 872 10.1038/ki.2008.350 18633339

[B21] Egger U Blumberg A Marti HR Acid–base balance and oxygen affinity of hemoglobin in patients on maintenance dialysis Clin Nephrol 1973 1 2 70 75 4669789

[B22] Klaus S Heringlake M Gliemroth J Pagel H Staubach K Bahlmann L Biochemical tissue monitoring during hypoxia and reoxygenation Resuscitation 2003 56 3 299 305 10.1016/S0300-9572(02)00342-8 12628561

[B23] Yee Koh M Spivak-Kroizman TR Powis G HIF-1 regulation: not so easy come, easy go Trends Biochem Sci 2008 33 11 526 534 10.1016/j.tibs.2008.08.002 18809331

[B24] Higgins DF Kimura K Bernhardt WM Shrimanker N Akai Y Hohenstein B Saito Y Johnson RS Kretzler M Cohen CD Eckardt KU Iwano M Haase VH Hypoxia promotes fibrogenesis in vivo via HIF-1 stimulation of epithelial-to-mesenchymal transition J Clin Invest 2007 117 3810 3820 18037992 10.1172/JCI30487 PMC2082142

[B25] Neusser MA Lindenmeyer MT Moll AG Segerer S Edenhofer I Sen K Stiehl DP Kretzler M Gröne HJ Schlöndorff D Cohen CD Human nephrosclerosis triggers a hypoxia-related glomerulopathy Am J Pathol 2010 176 12 594 607 20019191 10.2353/ajpath.2010.090268 PMC2808068

[B26] Chiang CK Tanaka T Inagi R Fujita T Nangaku M Indoxyl sulfate, a representative uremic toxin, suppresses erythropoietin production in a HIF-dependent manner Lab Invest 2011 91 11 1564 1571 10.1038/labinvest.2011.114 21863063

[B27] Chiang CK Tanaka T Nangaku M Dysregulated oxygen metabolism of the kidney by uremic toxins: review J Ren Nutr 2012 22 1 77 80 10.1053/j.jrn.2011.10.028 22200419

[B28] Sun S Du R Xia L Sun W Zhai Y Yu Y Zhao A Huang C Ning X Wang H Twist is a new prognostic marker for renal survival in patients with chronic kidney disease Am J Nephrol 2012 35 2 141 151 10.1159/000335191 22248584

[B29] Hadi BA Al-jubouri RH Salivary and plasma analysis of oxidative stress biomarkers in end stage renal failure patients J Bagh Coll Dentistry 2011 23 2 46 50

[B30] Sommerburg O Grune T Ehrich JH Siemens WG Adaptation of glutation-peroxidase activity to oxidative stress occurs in children but not in adult patients with end-stage renal failure undergoing hemodialysis Clin Nephrol 2000 58 1 S31 S36 12227724

[B31] Elshamaa MF Sabry S Nabih M Elghoroury EA El-Saaid GS Ismail AAG Alteration in plasma total antioxidant capacity, cardiotoxic lipid peroxidation product and C-reactive protein: a possible explanation for the increased cardiovascular risk in children on hemodialysis J Clin Basic Cardiol 2008 11 1–4 2 7

[B32] Turi S Varga I Matkovics B Dobos E Erythrocyte defence mechanisms against free oxygen radicals in haemodialysed uraemic children Pediatr Nephrol 1991 5 2 179 183 10.1007/BF01095947 2031830

[B33] Daschner M Lenhartz H Botticher D Schaefer F Wollschlager M Mehls O Leichsenring M Influence of dialysis on plasma lipid peroxidation products and antioxidant levels Kidney Int 1996 50 4 1268 1272 10.1038/ki.1996.437 8887287

[B34] Samouilidou EC Grapsa EJ Kakavas I Lagouranis A Agrogiannis B Oxidative stress markers and C-reactive protein in end-stage renal failure patients on dialysis Int Urol Nephrol 2003 35 3 393 397 15160547 10.1023/b:urol.0000022846.83505.3f

[B35] Pawlak K Pawlak D Mysliwiec M Oxidative stress influences CC-chemokine levels in hemodialyzed patients Nephron Physiol 2004 96 4 105 112 10.1159/000077381 15122056

[B36] Pupim LB Himmelfarb J McMonagle E Shyr Y Ikizler TA Influence of initiation of maintenance hemodialysis on biomarkers of inflammation and oxidative stress Kidney Int 2004 65 6 2371 2379 10.1111/j.1523-1755.2004.00656.x 15149350

[B37] Dursun E Dursun B Suleymanlar G Ozben T Effect of haemodialysis on the oxidative stress and antioxidants in diabetes mellitus Acta Diabetol 2005 42 3 123 128 10.1007/s00592-005-0191-1 16258735

[B38] Pavlova EL Lilova MI Savov VM Oxidative stress in children with kidney disease Pediatr Nephrol 2005 20 11 1599 1604 10.1007/s00467-005-1990-x 16001281

[B39] Galli F Ghibelli L Buoncristiani U Bordoni V D’Intini V Benedetti S Canestrari F Ronco C Floridi A Mononuclear leukocyte apoptosis in haemodialysis patients: the role of cell thiols and vitamin E Nephrol Dial Transplant 2003 18 8 1592 1600 10.1093/ndt/gfg210 12897100

[B40] Schouten WE Grooteman MP Van Houte AJ Schoorl M van Limbeek J Nube MJ Effects of dialyser and dialysate on the acute phase reaction in clinical bicarbonate dialysis Nephrol Dial Transplant 2000 15 3 379 384 10.1093/ndt/15.3.379 10692524

[B41] Drai J Bannier E Chazot C Hurot JM Goedert G Jean G Charra B Laurent G Baltassat P Revol A Oxidants and antioxidants in long-term haemodialysis patients Il Farmaco 2001 56 5–7 463 465 11482779 10.1016/s0014-827x(01)01063-1

[B42] Gerardi G Usberti M Martini G Albertini A Sugherini L Pompella A Di LD Plasma total antioxidant capacity in hemodialyzed patients and its relationships to other biomarkers of oxidative stress and lipid peroxidation Clin Chem Lab Med 2002 40 2 104 110 11939481 10.1515/CCLM.2002.019

[B43] Samouilidou E Grapsa E Effect of dialysis on plasma total antioxidant capacity and lipid peroxidation products in patients with end-stage renal failure Blood Purif 2003 21 3 209 212 10.1159/000070691 12784045

[B44] Haigis MC Deng CX Finley LW Kim HS Gius D SIRT3 Is a mitochondrial tumor suppressor: a scientific tale that connects aberrant cellular ROS, the warburg effect, and carcinogenesis Cancer Res 2012 72 10 2468 2472 10.1158/0008-5472.CAN-11-3633 22589271 PMC3354726

